# Long-read sequencing of oropharyngeal squamous cell carcinoma tumors reveal diverse patterns of high-risk Human Papillomavirus integration

**DOI:** 10.3389/fonc.2023.1264646

**Published:** 2023-10-17

**Authors:** Marc-Andre Gauthier, Adway Kadam, Gary Leveque, Nahid Golabi, Anthony Zeitouni, Keith Richardson, Marco Mascarella, Nader Sadeghi, Sampath Kumar Loganathan

**Affiliations:** ^1^ Department of Otolaryngology, Head and Neck Surgery, Faculty of Medicine, McGill University, Montreal, QC, Canada; ^2^ Cancer Research Program, Research Institute of the McGill University Health Centre, Montreal, QC, Canada; ^3^ Department of Experimental Surgery, Faculty of Medicine, McGill University, Montreal, QC, Canada; ^4^ Canadian Centre for Computational Genomics, McGill University, Montreal, QC, Canada; ^5^ Department of Human Genetics, McGill University, Montreal, QC, Canada; ^6^ Department of Oncology, McGill University, Montreal, QC, Canada; ^7^ Department of Experimental Medicine, Department of Biochemistry and Goodman Cancer Research Institute, McGill University, Montreal, QC, Canada

**Keywords:** HPV, OPSCC, long-read sequencing, HPV integration, nanopore, HNSCC

## Abstract

**Introduction:**

In North America and in most European countries, Human Papillomavirus (HPV) is responsible for over 70% of oropharyngeal squamous cell carcinomas. The burden of OPSCC, in high-income countries, has been steadily increasing over the past 20 years. As a result, in the USA and in the UK, the burden of HPV-related oropharyngeal squamous cell carcinoma in men has now surpassed that of cervical cancer in women. However, the oncogenic impact of high-risk HPV integration in oropharyngeal squamous cell carcinomas hasn’t been extensively studied. The present study aimed to explore the patterns of HPV integration in oropharyngeal squamous cell carcinomas and to assess the feasibility and reliability of long-read sequencing technology in detecting viral integration events in oropharyngeal head and neck cancers.

**Methods:**

A cohort of eight HPV-positive OPSCC pre-treatment patient tumors (four males and four females), were selected. All patients received a p16^INK4A^ positive OPSCC diagnosis and were treated at the McGill University Health Centre, a quaternary center in Montreal. A minimum of 20mg of tumor tissue was used for DNA extraction. Extracted DNA was subjected to Nanopore long-read sequencing to detect and analyze for the presence of high-risk HPV sequences. PCR and Sanger sequencing experiments were performed to confirm Nanopore long-read sequencing readings.

**Results:**

Nanopore long-read sequencing showed that seven out of eight patient samples displayed either integrated or episomal high-risk HPV sequences. Out of these seven samples, four displayed verifiable integration events upon bioinformatic analysis. Integration confirmation experiments were designed for all four samples using PCR-based methods. Sanger sequencing was also performed. Four distinct HPV integration patterns were identified: concatemer chromosomal integration in a single chromosome, bi-chromosomal concatemer integration, single chromosome complete integration and bi-chromosomal complete integration. HPV concatemer integration also proved more common than full HPV integration events.

**Conclusion and relevance:**

Long-read sequencing technologies can be effectively used to assess HPV integration patterns in OPSCC tumors. Clinically, more research should be conducted on the prognostication value of high-risk HPV integration in OPSCC tumors using long-read sequencing technology.

## Introduction

Oropharyngeal Squamous Cell Carcinoma (OPSCC) is a subtype of Head and Neck Squamous Cell Carcinoma (HNSCC) that typically arises from 4 main subsites: base of tongue (BOT), tonsils, soft palate, and posterior pharyngeal wall (1). In North America and most European countries, Human Papillomavirus (HPV) is responsible for over 70% of OPSCC cases (2, 3). HPV-related OPSCC predominantly affects Caucasian (90%) males (87%) between the age of 50 to 65 years of age (3–6). The burden of OPSCC, in high-income countries, has been steadily increasing over the past 20 years (3). As a result, in the USA and in the UK, the burden of HPV-positive OPSCC in men has now surpassed that of cervical cancer in women (1). The rise in HPV-positive OPSCC incidence presents a lot of challenges. Currently, gender-neutral vaccination efforts against HPV are still at an early stage and vaccine intake and indications vary across different national and international jurisdictions (7, 8). Comprehensive understanding of the impact of HPV vaccination programs on the epidemiology of OPSCC is not expected until 2050 (9, 10). In this setting, the increasing burden of HPV-related OPSCCs warrants clinicians and scientists to further investigate this disease and its pathogenic mechanisms.

HPV is the most common sexually transmitted infection in the World (1). As of today, 14 high-risk mucosal strains have been identified (3). High-risk HPV-16 is responsible for over 85% of HPV-related OPSCC cases (1, 3). HPV viruses have a non-enveloped, circular double-stranded DNA genome of approximately 8 kilobase pairs (kb) (11). HPV is an epitheliotropic virus composed of early (E1, E2, E4, E5, E6, E7) and late genes (L1, L2). Early genes are known to be responsible for the reproductive phase of HPV’s life cycle. Whereas late genes are responsible for encoding the HPV capsid protein. HPV infection debuts when extrachromosomal episomes enter the nucleus of epithelial basal cells (11–14). Upon entering the nucleus of epithelial basal cells, E1 and E2 start to generate replication proteins to support HPV genome amplification (11–14). The process of HPV genome amplification is called rolling circle reparation. During this process, HPV uses enzymes from the host’s homologous recombination machinery to amplify its genome. The inappropriate expression of these enzymes during the cell cycle is notably known for generating genomic instability (12–14). As a result, genomic DNA breaks occur, forming linearized HPV genomes and HPV concatemers and introducing possible HPV integration sites within the host’s genome. The introduction of DNA breaks triggers the DNA damage response (DDR) (12). This process is heavily impacted by early genes E6 and E7. E7 and E6 respectively disrupt the DDR by inhibiting CDK inhibitors (p21, p27) and by degrading the tumor suppressor gene p53 (12–14). As a result, entry into the S-phase of the cell cycle is facilitated. E7 also inhibits the DDR by interacting with the ATR/CHK1 signalling axis, a checkpoint regulator of the G2 phase (12, 13). E6 and E7 are also involved in the maintenance and replication of HPV episomes (11, 12). Hence, dysregulation of the cell cycle and the DNA damage response by HPV’s early genes is the catalytic event required for persistent HPV infection.

Various studies have shown that the E2 gene is more commonly found in its episomal form. It is most active, early in the infectious process, as it represses highly carcinogenic E6 and E7 genes (11, 15). However, upon integration, later in the infectious process, the E2 gene is often lost (11–13). Furthermore, increased expression of E6 and E7 has also been associated with clonal integration of hrHPV. Hence, presence of E6 and E7 combined with the loss of E2 could be suggestive of hrHPV clonal integration. However, the mechanisms behind hrHPV clonal integration as well as its impact on the prognostic of OPSCC patients hasn’t been extensively studied.

HPV-positive OPSCCs have shown better overall survival and disease-free survival when compared with HPV-negative OPSCCs and all sites HNSCCs (16–18). As a result, numerous treatment de-intensification clinical trials have been looking into minimizing treatment toxicity while maximizing treatment efficacy in HPV-positive OPSCC patients (19). The AJCC’s most recent edition also included p16^INK4A^ positivity (IHC) into the staging of OPSCC tumors (20, 21). However, amidst the addition of the p16^INK4A^ surrogate marker in the staging of OPSCC patients, over 15% of patients continue to show recurrent disease and/or fail to respond to current treatment de-intensification strategies (1, 22). In this context, identifying prognostication factors using readily accessible technologies could help clinicians inch towards more personalized and safer treatment protocols for their patients.

Previous studies, using Next-Generation Sequencing (NGS), have shown that viral integration of hrHPV strains, namely HPV-16 and HPV-18, was associated with worse overall survival in HPV-positive OPSCC patients (23–26). Similar findings have also been reported in hrHPV cervical cancers (27, 28). NGS platforms have been used across various cancer types and have proven to be highly reliable and accurate for use in genomic studies (29). However, NGS requires higher coverage and generates shorter reads (100-500bp) which creates uncertainty when mapping longer viral integration sequences (29). In recent years, Oxford Nanopore Technologies (ONT) and Pacific Biosciences (PacBio) released their long-read sequencing platforms. Oxford Nanopore long-read sequencing technology allows reads of up to 1 Mb in a single read with an average of 5-15 kb per read while PacBio allows reads of up to 70 kb in a single read (30–32). We chose Oxford Nanopore long read sequencing as they can produce reads up to 1 Mb to detect all types of HPV integration events. Long-read sequencing has already proven to be relatively accurate (> 90% accuracy) in analyzing and processing long genomic sequences such as viral integration events in host genomes (32). In this study, we sought to determine whether ONT’s Nanopore is reliable in identifying clonal integration events in HPV-positive OPSCCs. We also tried to identify new patterns of HPV integration and compare HPV integration patterns in OPSCC with cervical cancer.

## Methods

### Patient selection & sample collection

This study was approved by the Institutional Review Board of the McGill University Health Centre (MUHC) (Montreal, Canada) (REB, Head and Neck Disease Data and Bio-Bank, MP-37-2019-4659). All subjects provided informed consent to participate in this study. All 8 OPSCC samples were collected from the McGill University Health Centre HNSCC biobank. All tumor tissues were obtained at the time of diagnosis prior to treatment initiation. Tumor samples were collected in RPMI media, supplemented with 10% FBS, and stored at -80 °C. Considerations for sample selection included sex, OPSCC subsite and availability of sufficient tumor tissue (>20mg). Clinical information was extracted from the MUHC’s hospital electronic records. HPV status was established using immunohistochemistry (IHC) to assess for p16^INK4A^ overexpression, a surrogate marker for HPV positivity (20).

### DNA extraction, library construction & nanopore sequencing

Genomic DNA was extracted from 20 to 60mg of the tumor tissues using Nanobind® tissue kit (PacBio). The extracted DNA was processed through short read elimination kit (Circulomics, PacBio). DNA concentration for each sample was measured using Nanodrop 3300 (Thermo Fisher Scientific). Sequencing libraries were constructed using Oxford Nanopore Technologies’ (ONT) ligation sequencing kit (SQK-LSK109). Prepared libraries were subsequently sequenced using the PromethION platform and 1D flow cell, containing protein pore R.9.4.1. Nanopore sequencing results were processed using ONT’s Guppy software (v6.2.7) (to convert current intensity values (in fast5 format) into nucleic acid sequences (in fastq format). To generate 10-fold coverage, two libraries were run sequentially on each 1D flowcell platform. PycoQC (v.2.5.2) was used to compute the metrics and perform quality control of the ONT sequencing data.

### Bioinformatics analysis of long-read sequencing data

To confirm the presence of HPV-16 sequences, raw nanopore reads, in fasta format, were cross-referenced with the HPV-16 genome (NCBI: GCF_000863945.3) using blat. Upon confirmation of HPV-16 presence, minimap2 (v2.24) was used to align fastq-pass nanopore reads on the human GRCh38 reference genome. Sambamba (v.0.8.1) was then used to convert.sam alignment files to.bam format and to create the.bam index file. Long-read structural variant caller, svim (v.2.0.0), was used to detect all insertions on the human reference genome (GRCh38) found using the minimap2 alignment.bam files previously generated. Detected insertion sequences were then processed using blastn megablast (v.2.10.0+) to solely identify HPV-16 positive insertion reads. The Integrative Genomics Viewer (IGV) was used to load alignment.bam files and ascertain the location of insertion events within the GRCh38 reference genome. As we employed short read elimination kit to enrich our DNA samples for long-length sequences, we applied 1 kb cut off while analysing the long-read sequencing data. Similar methodology was applied to identify HPV-18 positive insertion reads amongst all 8 patient samples and to identify any of the most common 200 HPV subtypes for patient HN0138 (PaVE database).

### PCR verification of HPV presence & integration events

Two sets of primers (forward and reverse) were ordered for each of the following 5 HPV genes: E2, E6, E7, L1, L2 (**Supplementary Table 1**). To design these primers, Human Papillomavirus type 16 (HPV16) complete genome (GenBank ID: K02718.1) was used as a template. Expected PCR product length varied from 100bp to 700bp approximately. All primers were tested for specificity using DNA extracted from HEK293T cells and no bands were detected (data not shown). Tumor sample without HPV sequence does not show any bands implying the specificity of the two sets of primers while showing bands for GAPDH primer control. New primers were then specifically designed for 4 representative integration events (1 per patient sample) based on ONT’s long-read sequencing sequences upstream and downstream of the integration event, such that one primer would bind to an integrated HPV genomic sequence, while the other binds to the human genome (**Supplementary Table 3**). Human GRch38/hg38 (GenBank ID: 883148) was used as a template for the human genome. Expected PCR product length varied from 400bp to 900bp approximately.

### Identify HPV episomes using exonuclease V

To establish the presence of circular HPV episomes in our patient tissue samples, approximately 50ng of DNA was digested using Exonuclease V (M0345S, NEB) for 1 hour at 37°C. 10ng of the digestion products and appropriate controls were then PCR-amplified using the following primer pairs: E6.2, and GAPDH. To verify the validity of the exonuclease reaction, an in-house plasmid pR26-HPV containing the HPV E6 and E7 genes was linearized using Kpn1 (R3142S, NEB) and Xho1 (R0146S, NEB) restriction enzymes. The 50ng of linearized and circular plasmids were then digested with Exonuclease V (M0345S, NEB) for 1 hour at 37°C. 10ng of these digested plasmids were then used as a template for PCR amplification with primer pairs E6.2 and E7.2 (33, 34).

### Confirmation of HPV integration events using sanger sequencing

PCR products were assessed using 2% agarose gel electrophoresis. PCR products were extracted using Monarch® DNA Gel Extraction Kit (T1020S, NEB). Purified products were subsequently sequenced using the Sanger sequencing platform. Results from Sanger sequencing data were then compared with ONT’s long-read sequencing data.

## Results

### Patient clinical information

All eight selected samples were p16-positive oropharyngeal cancers. Four tumor samples belonged to female patients and four belonged to male patients. Subsites included tonsils (4), base of tongue (BOT) (3) and soft palate (1). Patients were between 52 and 72 years of age at the time of diagnosis. Average and median age at diagnosis were respectively 61 and 62 years of age. Five out of eight patients had a significant smoking history (>10 pack-year). Three patients presented with stage I, four presented with stage 2 and one presented with stage 3 HPV-related oropharyngeal cancer. Follow-up durations varied between 6 to 60 months. As of today, none of these patients are deceased (**Table 1A**).

**Table 1:** **A)** Summary of clinical characteristics of the patient enrolled in this study.

Sample	Age	Sex	Subsite	Smoking	Staging (AJCC 8th edition)	Treatment	Follow up
HN0003	64	M	BOT	50 PY	T2N1M0	NECTORS	48 months
HN0026	53	F	Tonsil	10 PY	T2N1M0	NECTORS	60 months
HN0052	64	F	BOT	0 PY	T2N2M0	NECTORS	24 months
HN0072	52	M	Tonsil	0 PY	T4N2M0	CRT	24 months
HN0094	60	M	Tonsil	4 PY	T3N1M0	NECTORS	18 months
HN0138	64	M	Tonsil	25 PY	T3N1M0	SURGERY + ADJUVANT RT	18 months
HN0209	55	F	Soft Palate	15 PY	T3N0M0	CRT	6 months
HN0211	72	F	BOT	35 PY	T2N1M0	NEC + RT	6 months

NECTORS, NEOADJUVANT CHEMOTHERAPY WITH TRANSORAL ROBOTIC SURGERY; CRT, CONCURRENT CHEMORADIOTHERAPY; RT, RADIOTHERAPY; NEC, NEOAJDUVANT CHEMOTHERAPY.

**Table:** **B)** Long-read sequencing parameters for each patient sample showing the amount of patient tumor used to extract DNA, length and coverage of the reads.

Sample	Quantity (mg)	Reads	Average Length	Median Length	Coverage	HPV Events (>1Kb)	HPV Integration Events (>1Kb)
HN0003	25.8	3 343 303	8 763	5 827	8.95	7	0
HN0026	20.3	2 117 524	13 819	9 054	9.04	17	1
HN0052	60.7	3 218 965	9 017	3 986	9.00	25	0
HN0072	42.4	4 232 478	6 498	2 633	8.28	28	1
HN0094	34.2	6 642 746	4 847	2 421	9.87	9	7
HN0138	34.5	2 037 677	12 856	7 051	7.76	0	0
HN0209	50.4	3 324 184	9 441	4 205	9.43	2	0
HN0211	44.6	1 704 155	13 002	7 013	6.72	86	6

Using bioinformatic analysis, total HPV events above 1000 base pairs (both episomal and integration) were tabulated. NA, not applicable.

### Long-read sequencing analytics

Upon DNA extraction, samples were submitted for quality testing. All eight samples proved sufficient quantitatively and qualitatively. (**Supplementary Figure 1**; **Supplementary Table 2**). Median DNA coverage was 8.98X while the average DNA coverage was 8.63X. Median number of reads per sample was 3 271 575 with an average number of reads per sample of 3 327 629. Average read length varied between approximately 5000bp and 14000bp per read across all eight samples. Median read length also varied between approximately 2500bp and 9000bp per read across all our samples. A total of 174 HPV-16 reads, over 1000bp, were detected in our eight patient samples. HN0211 presented with the highest amount of HPV-16 reads with 86 (>1Kb) reads, while HN0138 presented with no HPV-16 reads. Other patient samples presented between 2 to 28 (>1Kb) HPV-16 reads. (**Table 1B**). All eight patient samples were also analyzed for HPV-18 presence, and none presented with HPV-18 sequences. HN0138 was submitted for further bioinformatic analysis. Upon alignment with the 200 most common HPV-strains, HN0138 continued to show no HPV-related sequences.

### PCR confirmation of HPV gene presence

**Figure 1:**
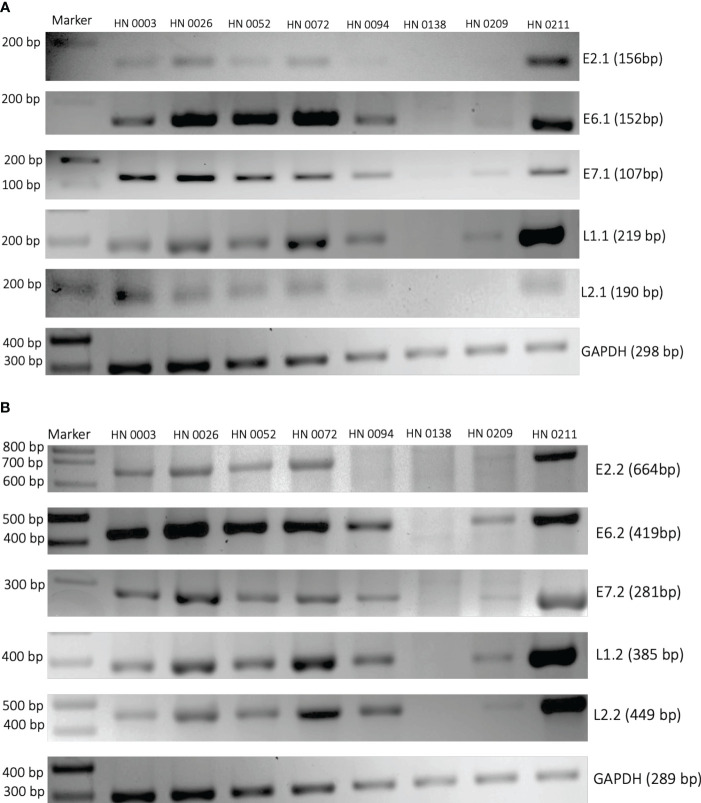
PCR amplification of HPV16 genes E2, E6, E7, L1 and L2 from patient tumor DNA. Two different sets of primers **(A)** First set and **(B)** Second set) were designed to target HPV16 genes. Patient tumors were used as the PCR template. Primers targeting human GAPDH were used as controls. Patient sample identification and expected band sizes are provided. 100 bp DNA ladder was used as a marker.

We designed two sets of reverse and forward primers for the following HPV genes: E2, E6, E7, L1, L2. Similar findings were made across both sets of primers for all five HPV genes. (**Figures 1A, B**). E2 gene could be found in six of the eight samples with HN0094 and HN0138 presenting no band on 2% agarose electrophoresis. While E6, E7, L1 and L2 genes could be found in seven of the eight samples with HN0138 being the only to show no presence of any of those genes on gel electrophoresis.

### PCR confirmation of HPV 16 integration

**Figure 2:**
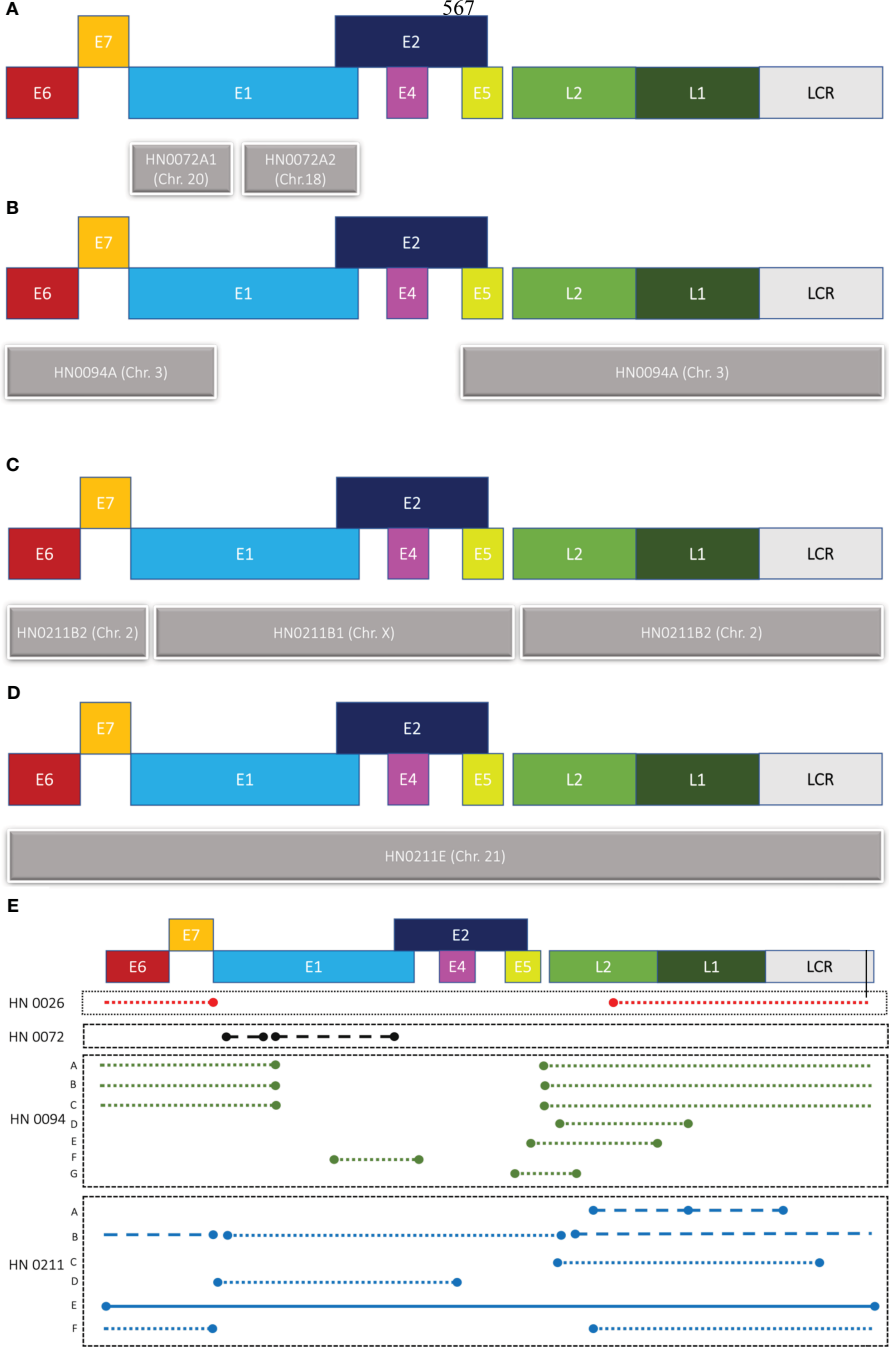
Graphical representation of HPV integration events. Different patterns of integration events are found in patient samples **(A)** HN0072, **(B)** HN0094 and **(C, D)** HN0211. **(E)** Representation of all HPV16 integration events detected by long-read sequencing across four samples (HN0026, HN0072, HN0094, HN0211). Concatemers can be found across three of our four samples, while full integration event could only be found in one patient sample: HN0211. Patient HN0094 also showed multiple readings of a single integration event. Close dots indicate concatemers on the same chromosome, while straight continuous line indicates the integration of the full HPV genome and dashed lines are concatemers that’s spread across multiple chromosomes. E1-7, L1 & 2 are HPV genes and LCR stands for Long Control region of HPV genome.

**Figure 3:**
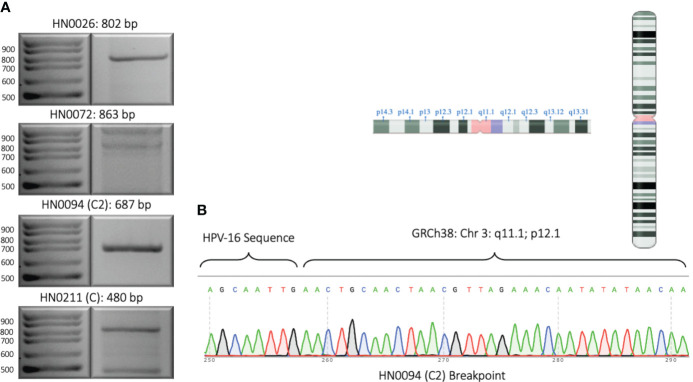
Experimental validation of representative HPV integration events. **(A)** 2% DNA agarose gel showing PCR amplified segments identified by long-read sequencing. One integration event for each sample was selected and primers were designed such that one primer anneals to the HPV genome and the other primer anneals to the human genome. Expected product size is provided. **(B)** Sanger sequencing of PCR amplified integration event from patient sample HN0094 showing the junction of HPV16 sequence and the human genome sequence. Inlet: the chromosome arm and location where the HPV16 sequence is integrated as revealed by long-read sequencing and confirmed by Sanger sequencing.

We designed one set of reverse and forward primers for each of the patient samples that presented with an integration upon bioinformatic analysis of long-read sequencing data. Out of all eight samples, four showed at least one integration event. (**Figure 2**; **Table 1B**). Patients HN0026, HN0072, HN0094 and HN0211 presented respectively 1, 1, 7 and 6 potential integration events spanning across the entire human genome. For samples with multiple reads, we selected the ONT read with the least base-pair mismatch to guide our primer selection. As a result, HN0094C2 and HN0211C were the integration events retained for PCR confirmation experiments, alongside HN0026 and HN0072’s lone integration event. (**Table 1B**). PCR confirmation analysis showed integration events for all four samples, confirming our long-read sequencing findings (**Figure 3A**). To confirm the ONT integration sequence, a representative PCR band from OPSCC 0094 tumor DNA was excised and Sanger sequenced to reveal the HPV genome and human genome junction (**Figure 3B**).

### Long-read sequencing integration analysis

Patient HN0138 never showed HPV-related events in long-read sequencing studies using ONT nor in PCR confirmation studies. The most likely reason for this finding is a false-positive p^16INK4A^ IHC test. Nonetheless, ONT’s long-read sequencing technology enabled us to accurately identify integration events across four out of seven samples (57%). These findings demonstrated heterogeneous integration patterns at diverse genomic breakpoints (**Table 2**). Patient HN0026 presented with a single concatemeric HPV integration event incorporating the E6 and E7 genes, without the E1 and E2 genes. HPV integration event was located on chromosome 20 within an intergenic region (**Table 2**). Patient HN0094 presented with several versions of the same HPV concatemeric event within an intergenic region on chromosome 3. All reads lacked the E2 gene and most presented with the E6 and E7 genes (**Figure 2A**; **Table 2**). Patient HN0072 also presented with concatemeric HPV integration. Patient presented with a short 1.5kbp integration event only comprising the E1 gene. Interestingly, this event spanned across two chromosomes, 18 and 20, and was located within intronic regions (**Figure 2B**; **Table 2**). Upon further analysing this event, the single long-read sequence contained concatemeric HPV integration followed by ~3.7kbp of chromosome 18 sequence and then ~77kbp of chromosome 20 sequence indicating the possibility that chromosome 18 got inserted in this location. Patient HN0211 presented with very diverse patterns of HPV integration. The HN0211(A) sequence presented as two HPV concatemeric events scattered across two chromosomes (9 and 22). This integration solely comprised the L1 and L2 genes. On the other hand, HN0211(B1) and HN0211(B2) presented complete HPV sequence, but concatemerized and dispersed across two chromosomes (X and 2). Complete integration event on a single chromosome (21) was also identified in this patient HN0211(E). Finally, concatemeric integration within a single chromosome was also identified in this patient HN0211(F). In which case, the patient presented with an integration event comprising E6 and E7, without the presence of the E2 gene (**Figures 2C, D**; **Table 2**).

**Table 2:** Bioinformatic report of HPV integration events identified using long-read sequencing.

Sample	Identification	Chromosome	Nanopore Read Start	Nanopore Read End	Length of integration	Region	Genes	Distance from closest gene
HN0026	1bf2fe01	20	29679468	29672233	3183	Intergenic	NINL	4 Mb
HN0072A1	432a5dc5	20	19923982	20000437	1215	Exons + Introns	RIN2, NAA20, CRNKL1, CFAP61	NA
HN0072A2	432a5dc5	18	69675994	69672318	1215	Intron	DOK6	NA
HN0094A1	1a206db0_1	3	145475768	145461606	5403	Intergenic	PLSCR4	684 kb
HN0094A2	1a206db0_2	3	145475768	145461606	5403	Intergenic	PLSCR4	684 kb
HN0094B	2c66e3b7	3	145482876	145507367	5395	Intergenic	PLSCR4	684 kb
HN0094C1	d552dd38_1	3	145482949	145475958	5422	Intergenic	PLSCR4	684 kb
HN0094C2	d552dd38_2	3	145482949	145475958	5422	Intergenic	PLSCR4	684 kb
HN0094D	e3df6fff	NA	NA	NA	1667	NA	NA	NA
HN0094E	7c29281a	3	145476271	145475958	1564	Intergenic	PLSCR4	684 kb
HN0094F	6ac257c7	3	145474919	145475768	1186	Intergenic	PLSCR4	684 kb
HN0094G	f6e6a312	3	145475958	145476343	1182	Intergenic	PLSCR4	684 kb
HN0211A1	49317600	22	23136914	23130462	2137	Exons + Introns	GNAZ, RSPH14	NA
HN2011A2	49317600	9	29923666	29919530	2137	Intergenic	LINGO2	1.3 Mb
HN0211B1	67a77108_1	X	45663541	45707620	7949	Intergenic	DIPK2B	478 Kb
HN0211B2	67a77108_2	2	102924513	102915859	7949	Intron	TMEM182	NA
HN0211C	6e37c569	18	56721036	56717506	3343	Intron	WDR7	NA
HN0211D	7ef99502	7	31616473	31608734	2368	Intron	ITPRID1	NA
HN0211E	989b461e	21	9774117	9736168	7965	Intergenic	KCNE1B	2 Mb
HN0211F	ff8bb93d	1	210362754	210329529	2840	Intronic	HHAT	NA

The length of integration, the integration region in the human chromosome and the nearby genes are shown for each patient sample. Translocations, within mostly intronic regions, can be seen in patients HN0072 and HN0211. Patients HN0026 and HN0094 display single DNA breakpoint integration within exclusively intergenic regions.

### Detection of HPV integration events: using exonuclease V enzyme

**Figure 4:**
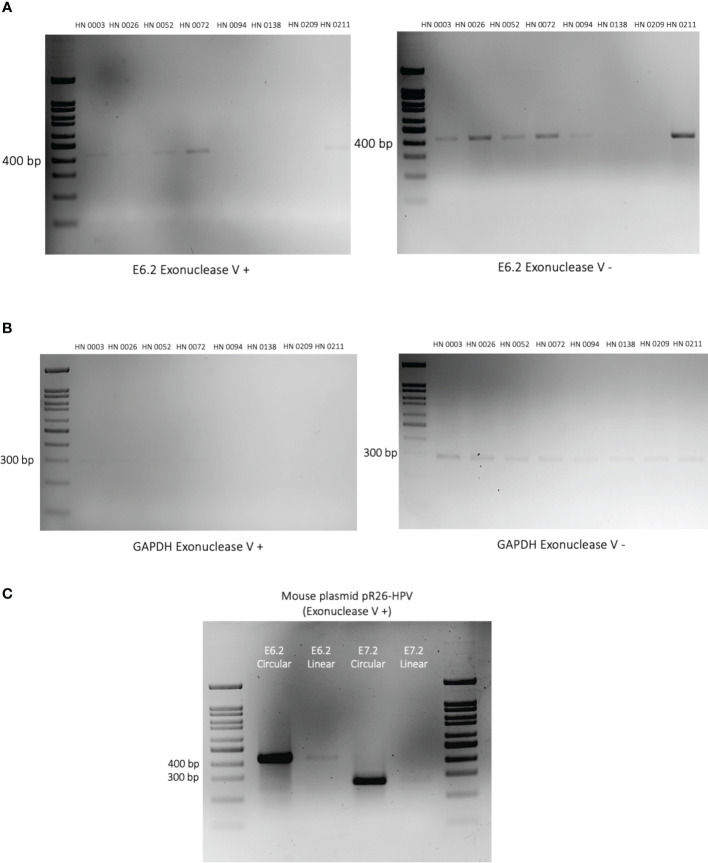
Episomal vs integration events in HPV-positive OPSCC patient tumors. **(A)** 2% DNA agarose gel showing the PCR amplified HPV E6 gene segment using Exonuclease V digested (left) and undigested (right) on patient DNA samples. Exonuclease V cleaves linear double-stranded DNA in both directions. Circular (episomal) DNA is resistant to Exonuclease V digestion. Patient identification is provided above each lane. **(B)** DNA agarose gel showing the PCR amplified human GAPDH gene segment in the presence (left) or absence (right) of Exonuclease V. GAPDH serves as a control as it is present in its linearized form and therefore sensitive to Exonuclease V digestion. **(C)** Plasmids containing HPV-E6/E7 oncogenes were linearized or left circularized and then treated with Exonuclease V. PCR amplification of both the E6 and E7 genes were performed to validate the Exonuclease V assay.

Exonuclease V is a DNA-specific exonuclease able to cleave linear single and double-stranded DNA strands into short oligos (<25 bp) (35). However, circular extrachromosomal episomes is resistant to this exonuclease (33). As a result, the intensity of the band, following exonuclease V exposure, can help us approximate the ratio of circular (episomal) to linear (integrated) HPV DNA found in our patient samples (34). For our experiment, we opted for our E6.2 primer set as it was precisely consistent across all samples. We also used linearized GAPDH and circular and linear versions of an in-house pR26-HPV mouse plasmid as controls. Our findings can be subdivided into three patterns of HPV integration vs episomal ratios: integration-dominant, episomal-dominant and mixed episomal-integration. Patient samples HN0138 and HN0209 did not show any convincing band in both Exonuclease V-negative and Exonuclease V-positive experiments. These findings are consistent with ONT findings, that identified respectively 0 and a mere 2 HPV-16 reads in these samples. Tumors HN0026, HN0094 and HN0211 presented with an integration-dominant pattern event. Tumors HN0003 and HN0052 presented with an episomal-dominant pattern. Whereas sample HN0072, presented with a mixed episomal-integration pattern (**Figure 4**).

## Discussion

In recent years, the expeditious rise in incidence of HPV-positive OPSCCs has presented healthcare professionals with serious epidemiological and clinical challenges (1–3). To address this situation, gender-neutral HPV vaccination programs have been established in most high-income countries where the burden of OPSCC is generally higher (7–9). Head and neck surgeons have also been actively involved in identifying better prognostication tools and safer treatment options to treat HPV-positive OPSCCs (19, 20). Amidst these efforts, up to 15% of HPV-positive OPSCC patients continue to show resistance to current treatment modalities (22, 35). For example, our clinical team at the McGill University Health Centre currently runs the NECTORS clinical trial. This trial combines neoadjuvant systemic treatment and locoregional treatment de-intensification for HPV-positive OPSCC patients to improve both oncologic and patient-reported quality of life outcomes (NCT04277858) (35–37). A fraction of OPSCC patients also continue to be misdiagnosed with HPV-related OPSCC due to the relatively moderate specificity (83%) of p16^INK4A^ (IHC) as a surrogate marker for HPV positivity (38). Other techniques like *in situ* hybridization (ISH), which allows the detection and localization of the viral genome within the cell and qRT-PCR of E6/E7 mRNA transcripts are available, but they are laborious and not feasible in clinical routine laboratories (38). Hence p16^INK4A^ (IHC) is routinely used to diagnose HPV even though there is 20 -30% chance of misdiagnosis. These HPV-related OPSCC misdiagnoses sometimes lead to unwarranted treatment de-escalation and result in poorer patient outcomes (1, 19). To address current challenges, we asked if we could use long-read sequencing technology to effectively identify HPV presence in OPSCC tumors and help us better understand patterns of HPV integration in the human genome.

Long-read sequencing studies on HPV-driven cancers, namely cervical cancer and OPSCC, remain scarce. In 2022, *Zhou et al.* performed long-read sequencing analyses on 16 cervical tumors and reported four distinct clonal integration patterns across all 16 samples. One of which, interestingly, did not include the highly oncogenic E6 and E7 genes into the integration events (31). *Yang et al.* also performed long-read sequencing on an HPV-35 positive cervical sample. Both studies reported that HPV-driven cervical cancers typically integrated the viral genome as concatemers (31, 34). Until recently, no study had investigated the patterns of HPV integration on OPSCC using long-read sequencing technology. However, in 2023, *Akagi et al.* performed long-read sequencing on five HPV positive OPSCC patient tumors. They identified a new type of genomic structural variation, named “heterocateny”, described as heterogeneous, interrelated, and repetitive patterns of integrated and concatemerized virus and host DNA segments (30). According to this study, “heterocateny” would contribute to chromosomal instability and rearrangement and promote tumorigenesis in OPSCCs (30). All three studies helped deepen our understanding of oncogenic mechanisms behind persistent hrHPV infection. More importantly, they also helped demonstrate the reliability of long-read sequencing technologies in studying virus-driven oncogenesis.

hrHPV integration has been associated with worse overall survival (OS) in HPV-positive OPSCC. In 2017, *Koneva et al.* used NGS to identify cases of hrHPV integration in 64 OPSCC samples. Out of 64 patient samples, 34 OPSCC patients (53%) displayed hrHPV integration (23). Overall survival probability amongst patients without HPV integration also proved significantly better, even after 8-year follow-up. Multivariate regression models to account for sex, subsite, clinical staging, smoking status, and age were also performed. Similar findings have also been reported amongst cervical cancer patient studies using NGS technologies (23). Furthermore, in 2022, *Stepp et al.* studied the usefulness of the NanoString gene differential expression assay to differentiate HPV integration from HPV episomal events in an HPV-positive OPSCC cohort (39). By demonstrating the reliability of this RNA-based assay, they successfully identified a more efficacious way to establish HPV integration status in HPV-positive OPSCC (39).

In this study, we sought to use ONT Nanopore long-read sequencing technology to help differentiate integration from episomal hrHPV events in HPV-positive OPSCCs and to identify new variations of HPV integration in OPSCC. To do so, we selected eight HPV-positive OPSCC patient tumors collected between 2019 and 2022. All eight tumors were classified as HPV-positive OPSCC based on p16^INK4A^ surrogate marker positivity. Long-read sequencing identified HPV sequences in seven of the eight samples (88%). Similar findings were found across PCR and exonuclease confirmation experiments. Hence, HN0138 was deemed a potential false positive HPV-positive OPSCC diagnosis. However, lack of DNA coverage in a patient presenting with a low viral load cannot be excluded. Presence of HPV-16, either episomal or integrated, was nonetheless confirmed in all seven remaining patient samples (**Figures 1A, B**). Amongst our seven truly HPV-positive OPSCC patients, four tumors (HN0026, HN0072, HN0094, HN0211) showed integration events (57%) upon long-read sequencing analysis. This is consistent with previous, NGS based studies, in both HPV-positive cervical and OPSCC cancers (22–26). These clonal integration events were confirmed by PCR-based integration experiments. (**Figure 3**). The validity of exonuclease V assays to discriminate HPV circular episomes from linearized integrated HPV has already been established in previous studies (33, 34). Correspondingly, three of these four integrated samples (HN0026, HN0094, HN0211) displayed an integration-dominant pattern. In contrast, HN0072 displayed an episomal-dominant pattern. However, episomal and integration events are not mutually exclusive events in HPV-driven carcinogenesis (1, 11, 14). Interestingly, HN0003 and HN0052 unveiled evidence of HPV integration in our exonuclease experiment. However, no integration events were identified during our long-read sequencing analysis. This incongruity could be explained by a lack of DNA sequencing coverage or low copy HPV DNA presence (29, 33). Interestingly, patients presenting with an integration-dominant did not present with later staging when compared with episomal-dominant or mixed episomal-integration patterns (p<0.001). However, our study wasn’t powered (n=8) enough to comprehensively address this issue.

Furthermore, we also identified different variants of HPV integration. All four samples with integration had evidence of HPV concatemer integration. However, some of them extended across a single chromosome (HN0026, HN0094), while others extended across multiple chromosomes (HN0072, HN0211). Complete HPV integration presented in only one patient (HN0211) as either two HPV concatemers divided amongst two chromosomes (HN0211B1 & HN0211B2) or as a single integration event on a single chromosome (HN0211E). Further studies are warranted to establish whether bi-chromosomal interactions, within a single tumor, are the results of translocation events post-HPV integration or simply the result of linearized concatemers integrating at fragilized chromosomal sites following DDR dysregulation by HPV.

In this study, integration events, identified using Nanopore, presented mostly as HPV concatemers. However, these concatemeric events presented in very different ways. Tumors HN0026 and HN0094 presented with single chromosomal integration at intergenic breakpoints, while tumors HN0072 and HN0211 presented with bi-chromosomal integrations at mostly intronic breakpoints. Such findings suggest that there are potentially various ways in which hrHPV strains interact with the human genome at the basal epidermal layer to induce carcinogenesis in HPV-positive OPSCC.

### Limitations

Our study looked into 8 HPV-positive OPSCC patient tumors from a single quaternary center in Montreal. Most of our patient samples were relatively recent diagnoses (<24 months). Hence, prognostication and outcome studies could not be performed. Furthermore, more patient samples would be needed, in the future, to truly establish prognostic and outcome values based on patterns of HPV-integration in OPSCCs. Finally, while our DNA coverage is within acceptable limits, access to higher DNA coverage could have further strengthen our data.

## Conclusion

Identification of HPV integration patterns in OPSCC, using long-read sequencing technology, hasn’t been extensively studied. This study helps confirm that long-read sequencing technology, namely Nanopore, can be effectively and reliably used to assess HPV integration patterns in OPSCC tumors. Four distinct HPV integration patterns were identified: concatemer chromosomal integration in a single chromosome, bi-chromosomal concatemer integration, single chromosome complete integration and bi-chromosomal complete integration. HPV concatemer integration (16) also proved more common than full HPV integration events (2). From a clinician’s point of view, more research should be conducted on the prognostication value of hrHPV integration in OPSCC tumors, using long-read sequencing technology, to guide staging and treatment de-intensification of HPV-positive OPSCC.

## Data availability statement

The datasets presented in this article are not readily available because of patient confidentiality and privacy. Requests to access the datasets should be directed to corresponding author SL.

## Ethics statement

The studies involving humans were approved by Research Ethics Board of McGill University Health Centre (REB, Head and Neck Disease Data and Bio-Bank, MP-37-2019-4659). The studies were conducted in accordance with the local legislation and institutional requirements. The participants provided their written informed consent to participate in this study.

## Author contributions

SL: Conceptualization, Funding acquisition, Investigation, Project administration, Resources, Supervision, Writing – original draft, Writing – review & editing. MG: Data curation, Formal Analysis, Methodology, Writing – original draft, Writing – review & editing. AK: Data curation, Investigation, Methodology, Writing – review & editing. GL: Formal Analysis, Software, Writing – review & editing. NG: Resources, Writing – review & editing. AZ: Resources, Writing – review & editing. KR: Resources, Writing – review & editing. MM: Resources, Writing – review & editing. NS: Funding acquisition, Resources, Supervision, Writing – review & editing.
