# Supplementary material for: Long-read sequencing of oropharyngeal squamous cell carcinoma tumors reveal diverse patterns of high-risk Human Papillomavirus integration
Source: Front Oncol. 2023 Oct 17;13:1264646. doi: 10.3389/fonc.2023.1264646 (PMC10616875; doi:10.3389/fonc.2023.1264646)
Supplement: Supplementary file 1 [file DataSheet_1.docx]

Supplementary Material

LONG-READ SEQUENCING OF OROPHARYNGEAL SQUAMOUS CELL CARCINOMA TUMORS REVEAL DIVERSE PATTERNS OF HIGH-RISK HUMAN PAPILLOMAVIRUS INTEGRATION

**Marc-Andre Gauthier MD^1,2^, Adway Kadam MSc ^2,3^, Gary Leveque MSc ^4,5^, Nahid Golabi MSc^1,2^, Anthony Zeitouni MD^1^, Keith Richardson MD^1^, Marco Mascarella MD^1,2^, Nader Sadeghi MD^1,2^, and Sampath Kumar Loganathan PhD^1,2,3,6,^ ***

*** Correspondence:**Sampath Kumar Loganathan, Cancer Research Program, RI-MUHC 1001 Decarie Boulevard, Montreal, QC, Canada H4A 3J1. ([sampath.loganathan@mcgill.ca](mailto:sampath.loganathan@mcgill.ca))

**SUPPLEMENTARY FIGURES**

**
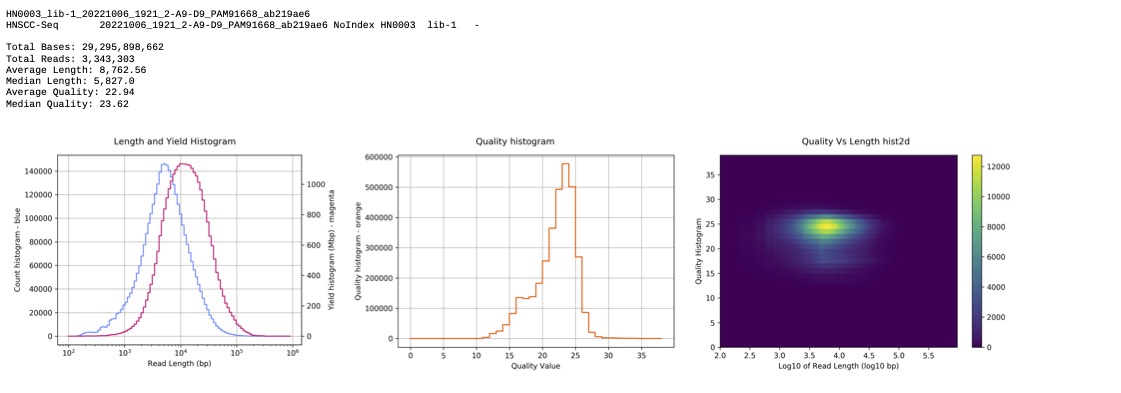

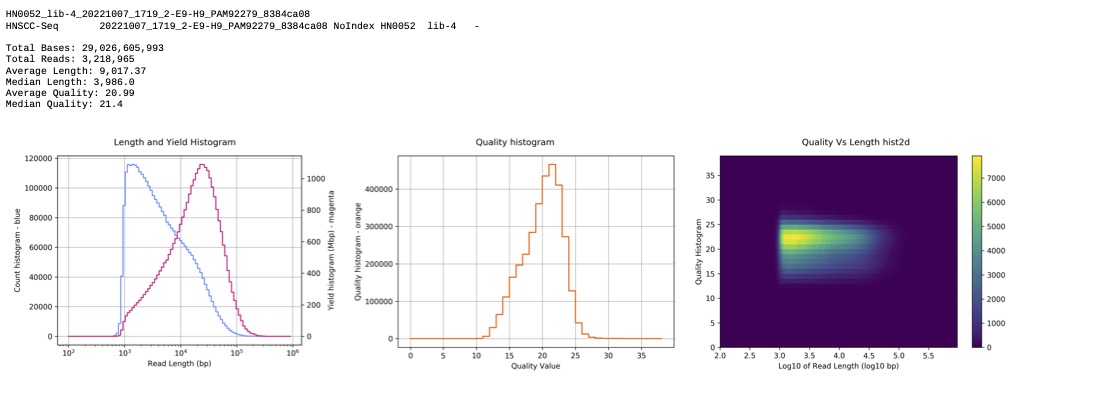

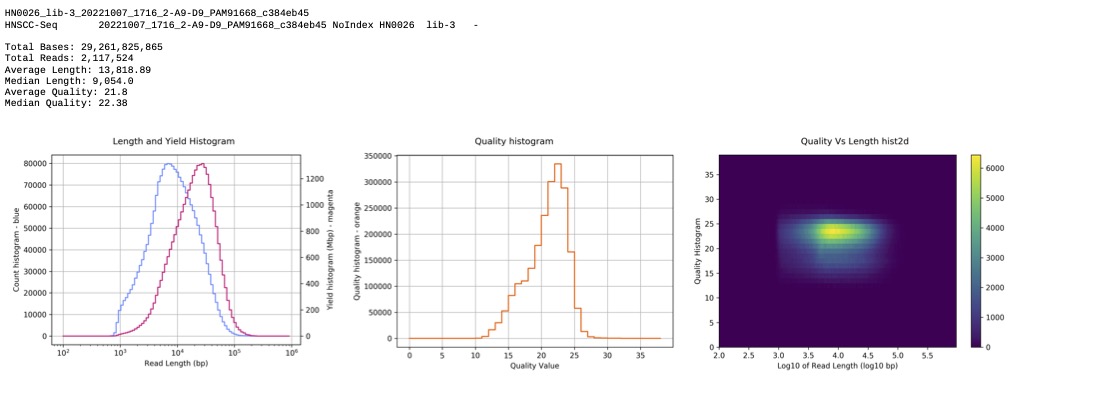
**

**
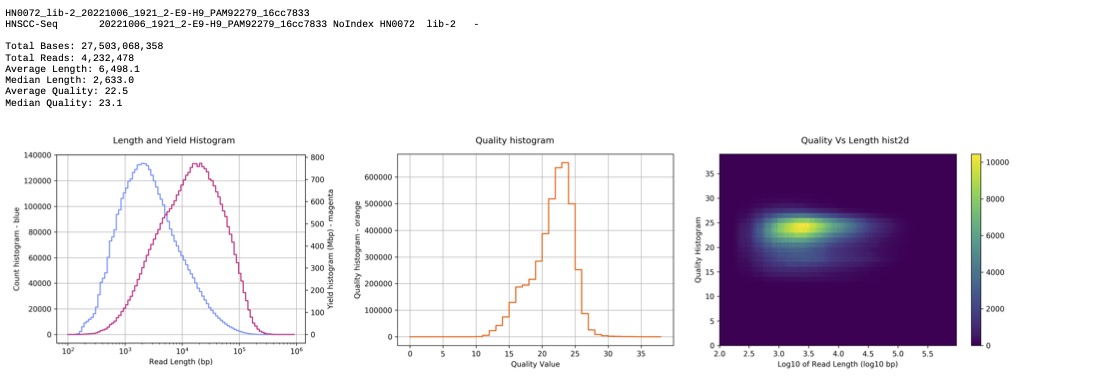

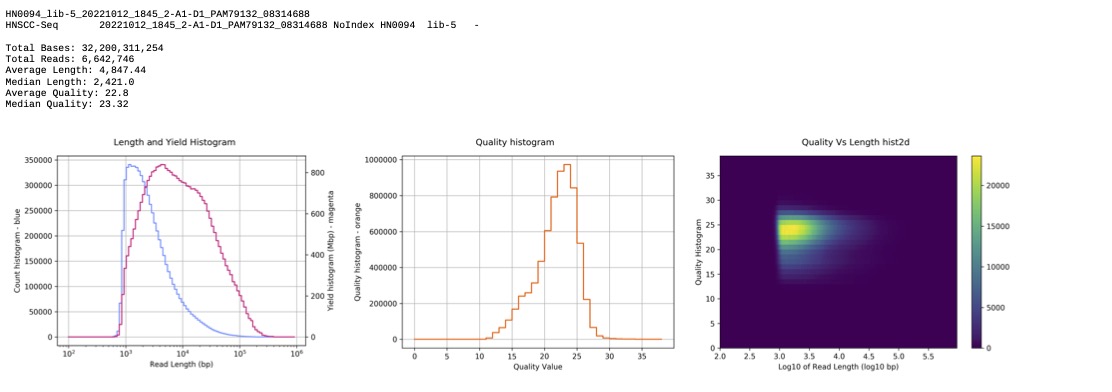

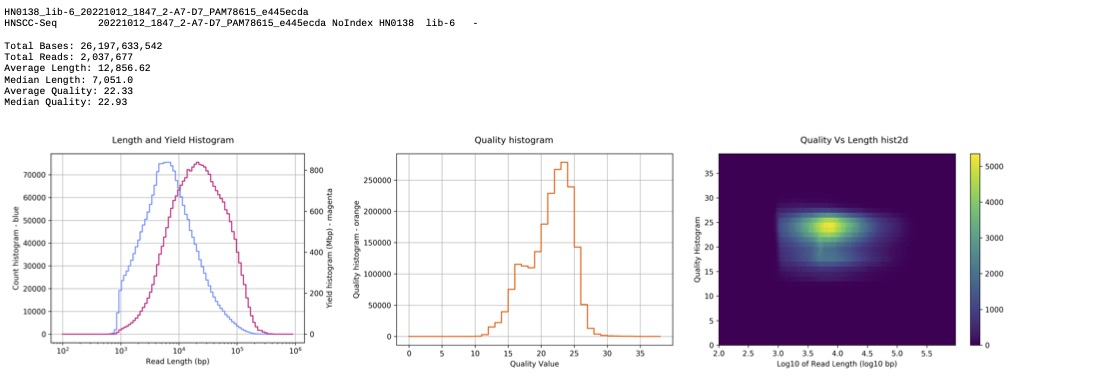
**

**
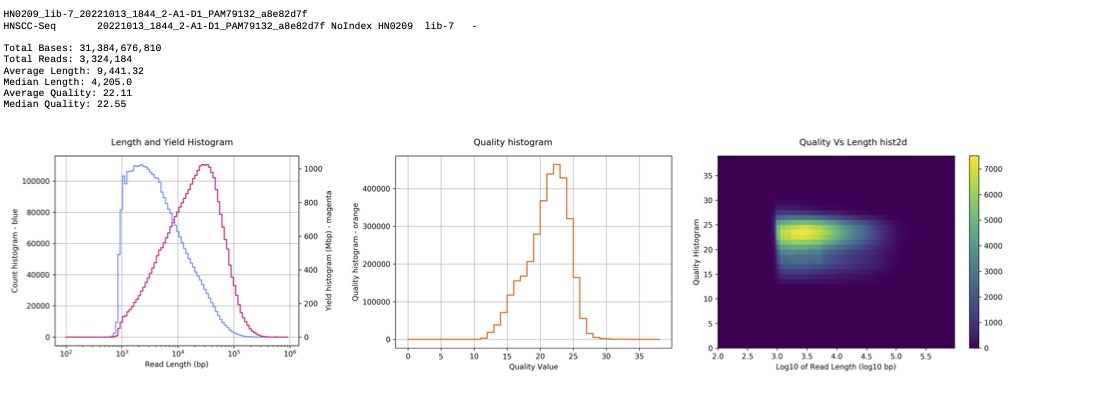

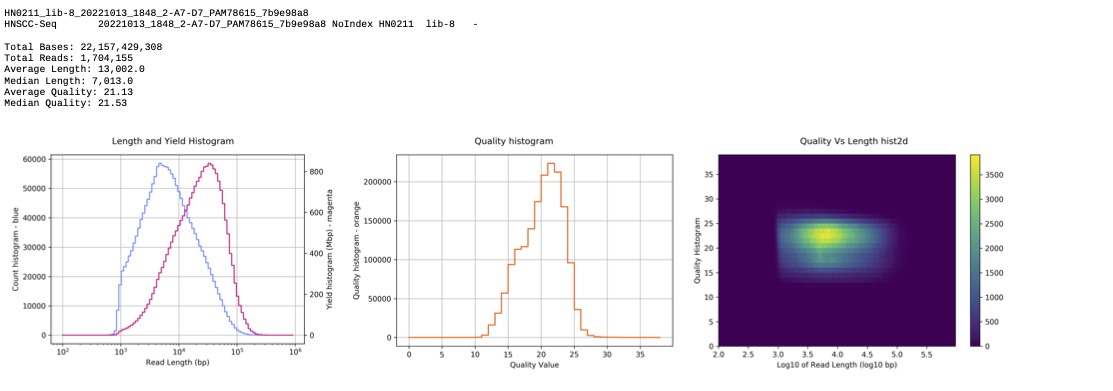
**

**Supplementary Figure 1:** Quality control parameters of the DNA extraction of all eight patient tumors used for long-read sequencing. Sequencing quality, length and coverage for each tumor tissue are provided.

**
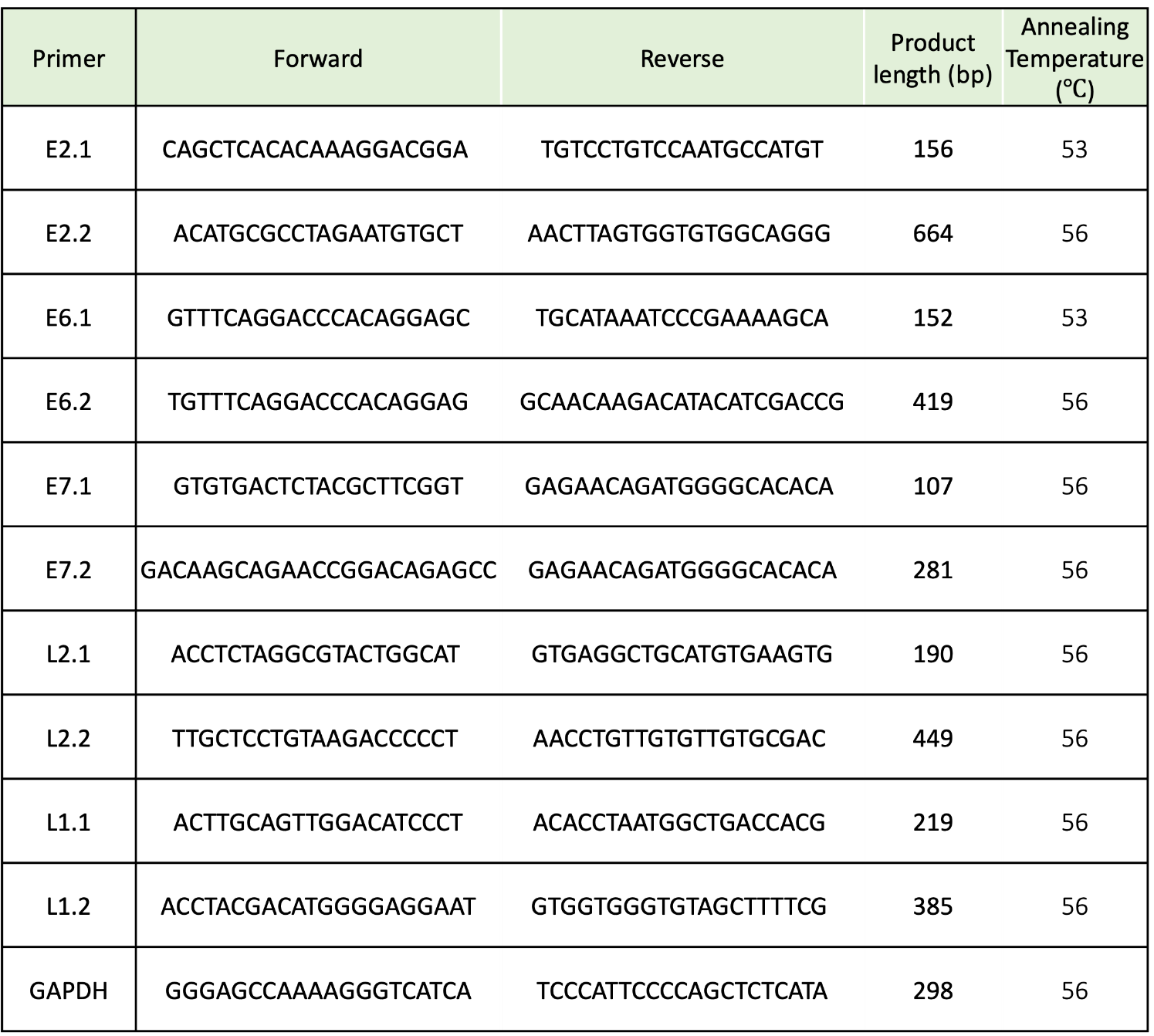
**

**Supplementary Table 1:** Primers used for PCR amplification of HPV-16 genes in respective patient samples. Two sets of primers were used to cover most areas of the HPV-16 genes. E2.1 represents the first set of primers for E2 while E2.2 represents the second set of primers for that same gene. Expected product sizes are also provided.


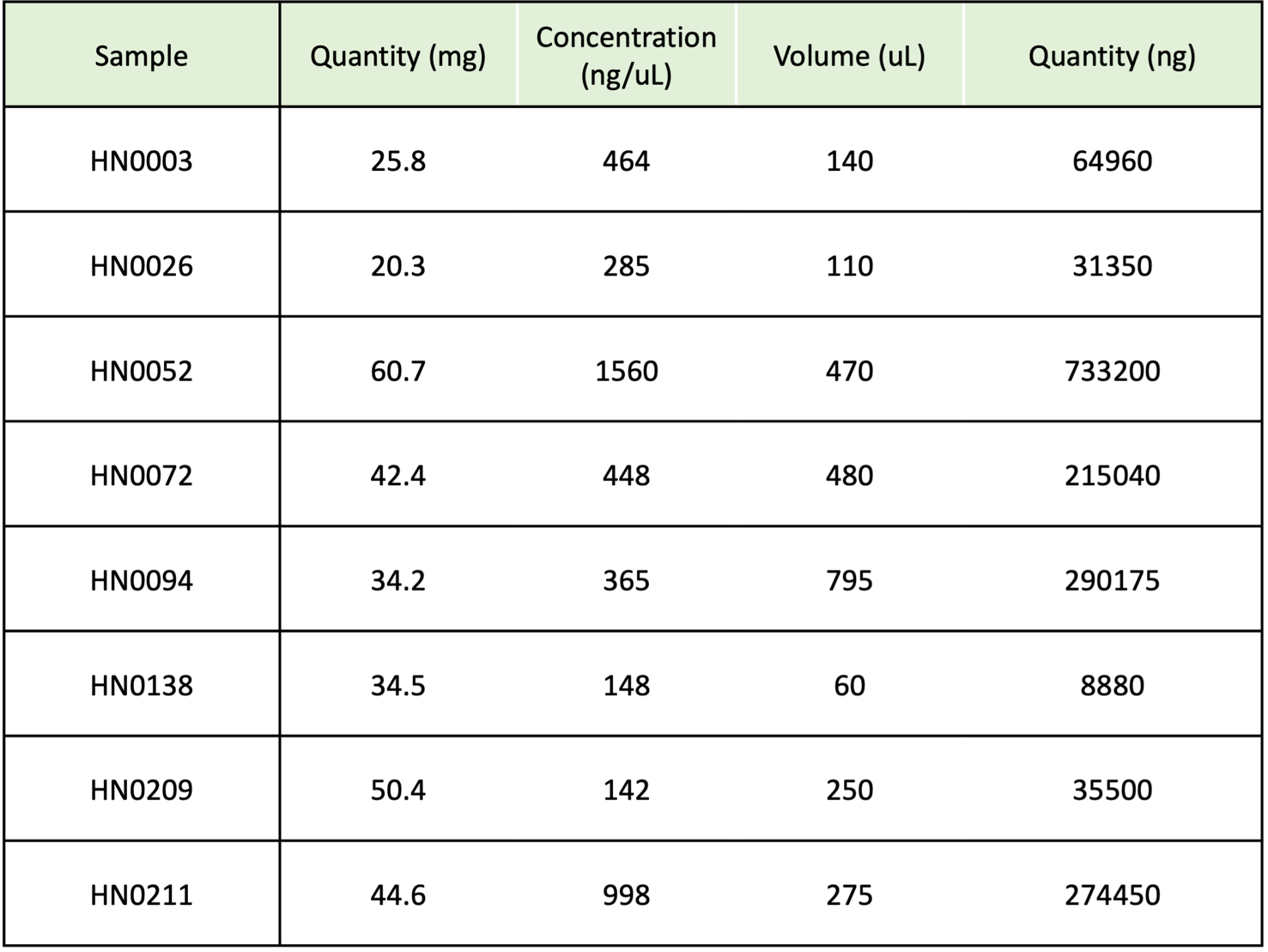


**Supplementary Table 2**: Sample characteristics used for long-read sequencing analysis. Amount of patient tumor used for DNA extraction, concentration and quantity of extracted DNA for each sample is provided.


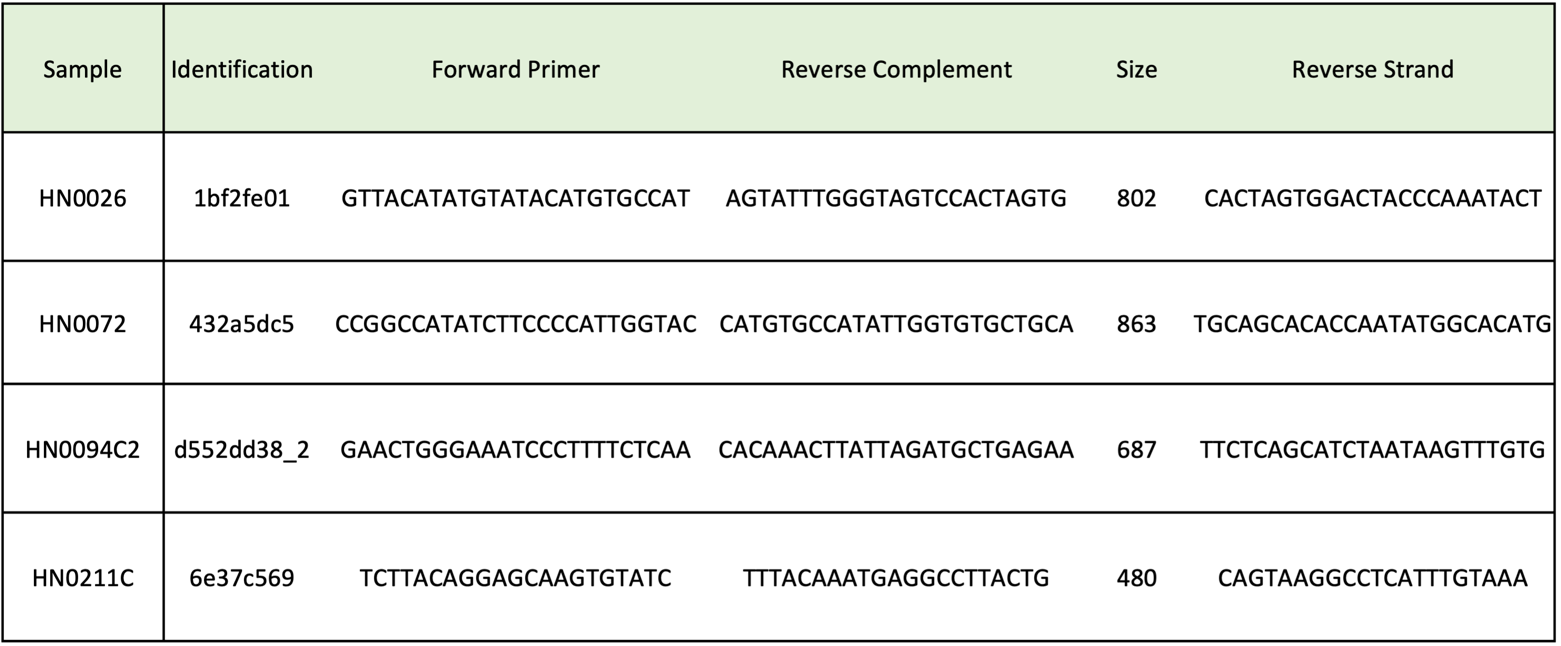


**Supplementary Table 3:** Primers used to amplify HPV integration sequence within the human genome. Primers were designed from long-read sequencing base pair sequences, such that one primer anneals to the HPV genome while the other primer anneals to the human genome.
